# Prognostic value and immune characteristics of RUNX gene family in human cancers: a pan-cancer analysis

**DOI:** 10.18632/aging.204065

**Published:** 2022-05-06

**Authors:** Han Zhao, Yun Chen, Peijun Shen, Lan Gong

**Affiliations:** 1 Department of Ophthalmology, Eye, Ear, Nose, and Throat Hospital of Fudan University, Shanghai 200000, Shanghai, China; 2 Laboratory of Myopia, NHC Key Laboratory of Myopia, Fudan University, Chinese Academy of Medical Sciences, Shanghai 200000, Shanghai, China; 3 Shanghai Key Laboratory of Visual Impairment and Restoration, Fudan University, Shanghai 200000, Shanghai, China; 4 Department of Stomatology, The Second Xiangya Hospital of Central South University, Changsha 410011, Hunan, China; 5 Department of Gastroenterology, The First Affiliated Hospital of Xinxiang Medical University, Weihui 453100, Henan, China

**Keywords:** runt-related transcription factors, pan-cancer, immune subtype, tumor microenvironment, prognoses

## Abstract

Background: Runt-related transcription factors (RUNX) are involved in numerous fundamental biological processes and play crucial parts in tumorigenesis and metastasis both directly and indirectly. However, the pan-cancer evidence of the *RUNX* gene family is not available.

Methods: In this study, we analyzed the potential association between *RUNX* gene family expression and patient’s prognosis, immune cell infiltration, drug response, and genetic mutation data across different types of tumors using based on The Cancer Genome Atlas, Gene Expression Omnibus, and Oncomine database.

Results: The results showed that the expression of the *RUNX* gene family varied among different cancer types, revealing its heterogeneity in cancers and that expression of RUNX2 was lower than that of RUNX1 and RUNX3 across all cancer types. *RUNX* gene family gene expression was related to prognosis in several cancers. Furthermore, our study revealed a clear association between *RUNX* gene family expression and ESTIMATE score, RNA stemness, and DNA stemness scores. Compared with RUNX1 and RUNX2, RUNX3 showed relatively low levels of genetic alterations. *RUNX* gene family genes had clear associations with immune infiltrate subtypes, and their expression was positively related to immune checkpoint genes and drug sensitivity in most cases. Two immunotherapy cohorts confirm that the expression of *RUNX* was correlated with the clinical response of immunotherapy.

Conclusions: These findings will help to elucidate the potential oncogenic roles of *RUNX* gene family genes in different types of cancer and it can function as a prognostic marker in various malignant tumors.

## INTRODUCTION

Runt-related transcription factors (RUNX) regulate many fundamental biological processes including proliferation, differentiation, apoptosis, and angiogenesis. According to structural analyses, *RUNX* gene family proteins is an evolutionarily conserved DNA-binding motif that dimerizes with core-binding factor subunit beta, a common non-DNA-binding partner. There are three *RUNX* genes in the human genome, *RUNX1*, *RUNX2*, and *RUNX3*, which show distinct expression patterns across human tissues. RUNX1 is essential for hematopoiesis and is involved in the development of human leukemia. RUNX1 is implicated in sensory neuron diversification in addition to its involvement in leukemogenesis [1, 2], RUNX1 is involved in sensory neuron diversification. RUNX2 is associated in skeletal mineralization and typically enhances mesenchymal differentiation of stem cells into osteoblasts, promotes chondrocyte hypertrophy, and aids endothelial cell migration and vascular penetration of growing bones [3]. RUNX3 regulates the differentiation and proliferation of gastrointestinal epithelial cells [4] and is found in hematopoietic cells such as myeloid and B-cell lines, as well as spleen cells [5].

In recent years, accumulating evidence has demonstrated that the *RUNX* gene family has crucial roles in tumor immunosuppression, determination of biological phenotypes, tumorigenesis, progression, metastasis, therapeutic effect, and patient prognosis, via both direct and indirect effects [6–9]. The *RUNX* gene family is intimately involved in carcinogenesis: the *RUNX1* gene was found to be mutated in human leukemia [10]; and RUNX2 is overexpressed in osteosarcoma and regulates bone remodeling and osteoclast differentiation [11]. Importantly, *RUNX2* is closely associated with drug resistance of various malignant cancers [12]. The *RUNX3* gene is located on chromosome 1p36, which is a hotspot depleted in various cancer types [13]. RUNX3 is expressed on various solid tumors and may contribute to tumor immunosuppression [14]. However, there is almost no data of the RUNX gene family playing a function in diverse tumor forms in humans.

Pan-cancer analysis may now be used to profile any gene of interest and its related clinical prognosis, as well as its probable molecular pathways in tumor and normal tissues, thanks to the rapid expansion of public databases. In this work, we used public datasets such as the Cancer Genome Atlas (TCGA) and Oncomine to undertake a pan-cancer analysis of RUNX gene family expression and patient prognosis across diverse forms of cancer. In addition, we present *RUNX* gene family profiles for immune cell infiltration, drug resistance, and genetic alterations to help doctors pick suitable treatment medications and enhance cancer patients’ prognoses.

## MATERIALS AND METHODS

### TCGA data and processing

The TCGA database (http://cancergenome.nih.gov) comprises the results of a groundbreaking cancer genomics study that has molecularly described over 20,000 primary cancer and normal samples encompassing 33 cancer types up to 2021. The TCGA data included 33 different tumor types: adrenocortical carcinoma (ACC); bladder urothelial carcinoma (BLCA); breast cancer (BRCA); cervical squamous cell carcinoma and endocervical adenocarcinoma; cholangiocarcinoma (CHOL); colon adenocarcinoma (COAD); lymphoid neoplasm diffuse large B-cell lymphoma; esophageal carcinoma (ESCA); glioblastoma multiforme (GBM); head and neck squamous carcinoma (HNSC); kidney chromophobe (KICH); kidney renal clear cell carcinoma (KIRC); kidney renal papillary cell carcinoma (KIRP); acute myeloid leukemia (LAML); brain lower grade glioma (LGG); liver hepatocellular carcinoma (LIHC); lung adenocarcinoma (LUAD); lung squamous cell carcinoma (LUSC); mesothelioma (MESO); ovarian serous cystadenocarcinoma (OV); pancreatic adenocarcinoma (PAAD); pheochromocytoma and paraganglioma (PCPG); prostate adenocarcinoma; rectum adenocarcinoma (READ); sarcoma (SARC); skin cutaneous melanoma (SKCM); stomach adenocarcinoma (STAD); testicular germ cell tumors; thyroid carcinoma (THCA); thymoma (THYM); uterine corpus endometrial carcinoma (UCEC); uterine carcinosarcoma; and uveal melanoma (UVM). The UCSC Xena (https://xenabrowser.net/) website was used to collect *RUNX* gene family data from TCGA, including RNA sequencing results, clinical data, stemness scores, DNA methylation, and immune subtype data.

### Gene expression data with immunotherapy

To investigate the predictive value of the *RUNX* gene family, two separate datasets, GSE78220 and IMvigor210, were obtained and examined. GSE78220 (platform GPL11154 Illumina HiSeq 2000) gene expression profiles and clinical data were acquired from GEO (https://www.ncbi.nlm.nih.gov/geo/) databases, which was chosen for this study as the anti-PD-1 immunotherapy cohort. The IMvigor210 dataset was obtained under the Creative Commons 3.0 license from a freely available, fully documented software and data package available, which was chosen for this study as the anti-PD-L1 immunotherapy cohorts.

### RUNX gene family expression analysis

In the TCGA, we utilized the “ggpubr” R package to compare the expression of *RUNX* gene family members in cancers and matching normal tissues. Gene expression was standardized in the pan-tumor study. Transcripts per kilobase million values were created by converting fragments per kilobase million values. Using the “pheatmap” R package, the findings were shown as heatmaps.

The Oncomine database (http://www.oncomine.org) has 715 datasets and 86,733 samples for genome-wide expression analysis [15]. We used Oncomine to compare the expression levels of *RUNX* gene family members in cancer tissues and neighboring normal tissues. In our research, we employed the student’s t-test to compare the expression of *RUNX* gene family members in different forms of cancer; a p-value of 0.05, a fold change of 2, and a gene rank in the top 10% were set as the significance thresholds.

### Survival prognosis analysis

The link of the RUNX gene family with overall survival (OS), disease-specific survival (DSS), progression-free interval (PFI), and disease-free interval (DFI) across all TCGA cancers was investigated using Kaplan–Meier curves. With 95 percent confidence intervals, we calculated log-rank p-values and hazard ratios (HR) (95 percent CI). Forest plots (created with the “forestplot” R package) and survival curves were used to visualize the data.

### Mutation profiles

The cBioPortal (https://www.cbioportal.org) website is an interactive tool for exploring, visualizing, and analyzing multidimensional cancer genomics data [16]. On a pan-cancer basis, we investigated the copy number changes and genetic modification features of the *RUNX* gene family using the “Cancer Types Summary” module. The “Cancer Types Summary” module of cBioPortal was used to acquire the alteration frequency, mutation type, and copy number alteration of the *RUNX* gene family across all tumors in TCGA. To query the genetic modification features of the *RUNX* gene family, we set “TCGA Pan Cancer Atlas Studies” in the “Quick pick” area.

### Tumor microenvironment and tumor stemness analysis

The “estimate” R package was used to measure tumor purity in 33 human malignancies from the TCGA. The ESTIMATE score is made up of the immunological and stromal scores, which indicate the amount of immune and stromal components in the body, respectively. Lower tumor purity is associated with higher ESTIMATE scores. C1 (wound healing), C2 [interferon (IFN)-r dominant], C3 (inflammatory), C4 (lymphocyte depletion), C5 (immunologically quiet), and C6 [tumor growth factor b (TGFb) dominant] are the six immune subtypes used to quantify immune infiltration in the tumor environment. The correlations between the expression of RUNX gene family members and the ESTIMATE immune and stromal scores were discovered using Spearman’s correlation analysis. We also used epigenetic and transcriptome data to calculate tumor RNA stemness scores (RNAss) and DNA stemness scores (DNAss). Specifically, RNAss is calculated using RNA sequencing data, whereas DNAss is calculated using DNA methylation data. The associations between the RUNX gene family and tumor stemness data were investigated using Spearman’s correlation analysis. The relationships between TGFBI expression and immunoinhibitory and immunostimulatory gene subsets, tumor mutation burden (TMB), and microsatellite instability were investigated using Pearson’s correlation analysis (MSI). Using R’s “pheatmap” package, the findings were visualized as heatmaps.

### Drug responses

The *RUNX* gene family medication response information were gathered from the National Cancer Institute (NCI)-60 database through the CellMiner database (https://discover.nci.nih.gov/cellminer/home.do). The NCI-60 database comprises molecular and pharmacological information for 60 different human cancer cell lines. Pearson’s correlation analysis was used to assess the relationship between *RUNX* gene family expression (as measured by transcript levels) and drug response (GI50). All of the medications used in the correlation study were identified by the Food and Drug Administration or through drug clinical studies.

### Statistical analyses

Oncomine was used to perform correlation analysis for the expression of *RUNX* gene family members between cancer and neighboring tissues, including p-values, fold changes, and gene rankings. R version 4.0.4, 64-bit (https://www.r-project.org/) and its associated packages were utilized in all studies. The outcomes of survival are displayed with HR, 95% CI, and log-rank *p*-values. For all statistical analyses, *p*<0.05 was considered statistically significant.

### Data availability statement

Publicly available datasets were analyzed in this study. This data can be found here: TCGA database (http://cancergenome.nih.gov), UCSC Xena (https://xenabrowser.net/), Oncomine database (http://www.oncomine.org), the National Cancer Institute (NCI)-60 database via the CellMiner database (https://discover.nci.nih.gov/cellminer/home.do), and GeneMANIA (http://www.genemania.org).

## RESULTS

### Gene expression analysis of RUNX

**Figure 1:**
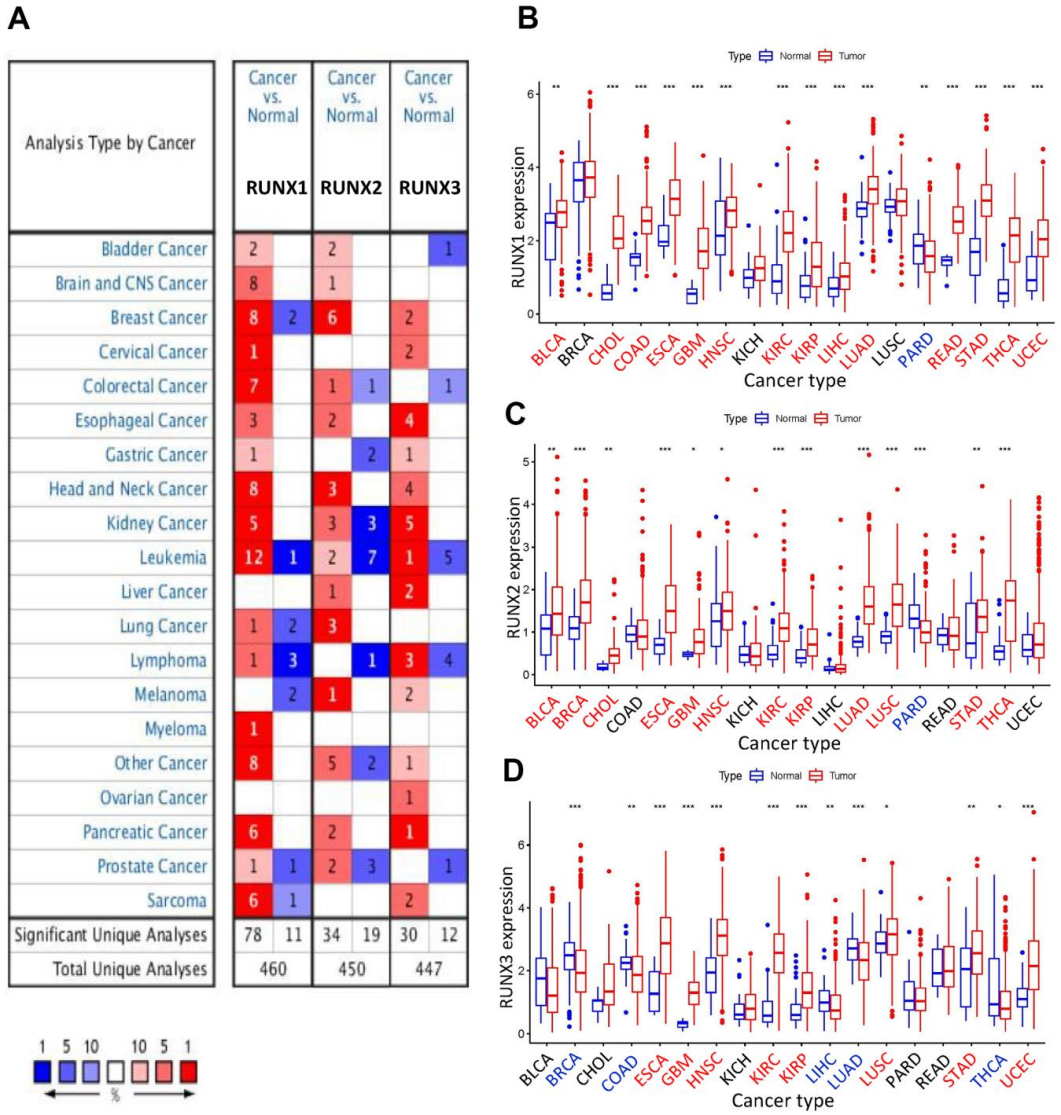
**Expression levels of *RUNX* gene family in different types of human cancers.** (**A**) Numbers of datasets with statistically significant increased (red) or decreased (blue) mRNA expression of RUNX from Oncomine. (**B**) RUNX1, (**C**) RUNX2, and (**D**) RUNX3 gene expression levels in different cancer types (red) and normal tissue (blue). *p<0.05, **p<0.01, and ***p<0.001. Names in red indicate high expression and those in blue indicate low expression of the corresponding RUNX gene family gene.

The mRNA levels of RUNX gene family members in malignancies and matched normal tissues were evaluated using the Oncomine database to compare expression levels between tumor and normal tissues. RUNX1 and RUNX2 expression was found to be quite high in head and neck cancer, kidney cancer, leukemia, and pancreatic cancer, according to our findings. RUNX1 was also shown to be greater in malignancies of the brain and central nervous system, colorectal cancer, and sarcoma. RUNX3 expression was shown to be greater in esophageal cancer, head and neck cancers, kidney cancer, lymphoma, and sarcoma than in other malignancies (Figure 1A).

Using the R program, we profiled and compared the expression of RUNX gene family members across all TCGA cancers to further analyze their differential expression. RUNX1 expression was considerably increased in BLCA, CHOL, COAD, ESCA, GBM, HNSC, LIHC, LUAD, READ, KIRC, KIRP, STAD, THCA, and UCEC, according to our findings. In prostate adenocarcinoma (PARD), however, RUNX1 expression was shown to be decreased (Figure 1B). RUNX2 expression was shown to be greater in BLCA, BRCA, CHOL, ESCA, GBM, HNSC, KIRC, KIRP, LUAD, LUSC, STAD, and THCA, but lower in PARD (Figure 1C). RUNX3 expression was shown to be substantially higher in ESCA, GBM, LUSC, HNSC, KIRC, KIRP, STAD, and UCEC than in BRCA, COAD, LIHC, LUAD, and THCA (Figure 1D).

**Figure 2:**
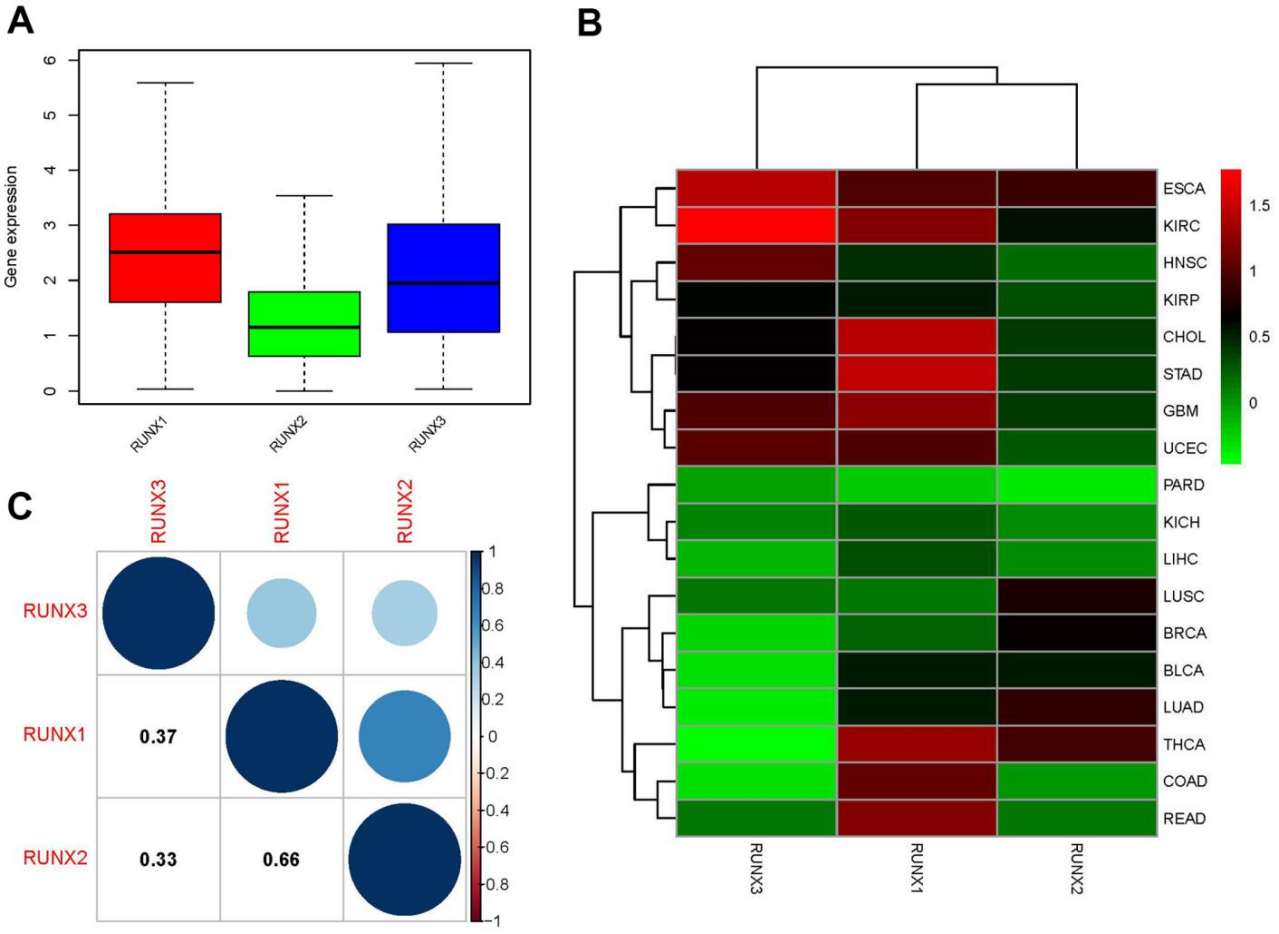
**Expression levels and correlations of *RUNX* gene family genes in different cancer types from TCGA.** (**A**) Boxplot of *RUNX* gene family gene expression across various cancer types. (**B**) Heatmap of *RUNX* gene family gene expression levels in different cancer types and normal tissues from TCGA data. (**C**) Positive (blue) and negative (red) correlations between *RUNX* gene family genes.

We also investigated at the pan-cancer and inter-tumor heterogeneity of *RUNX* gene family expression, and found that RUNX1 and RUNX3 were highly expressed at the pan-cancer level, whereas RUNX2 was modestly expressed (Figure 2A). We found considerable variation in gene expression of *RUNX* gene family members across various tumor types in TCGA, as illustrated in Figure 2B. Then we looked at the relationships between members of the *RUNX* gene family; RUNX1 and RUNX2 had the strongest positive association (correlation coefficient = 0.66, Figure 2C).

### Prognostic value of RUNX

**Figure 3:**
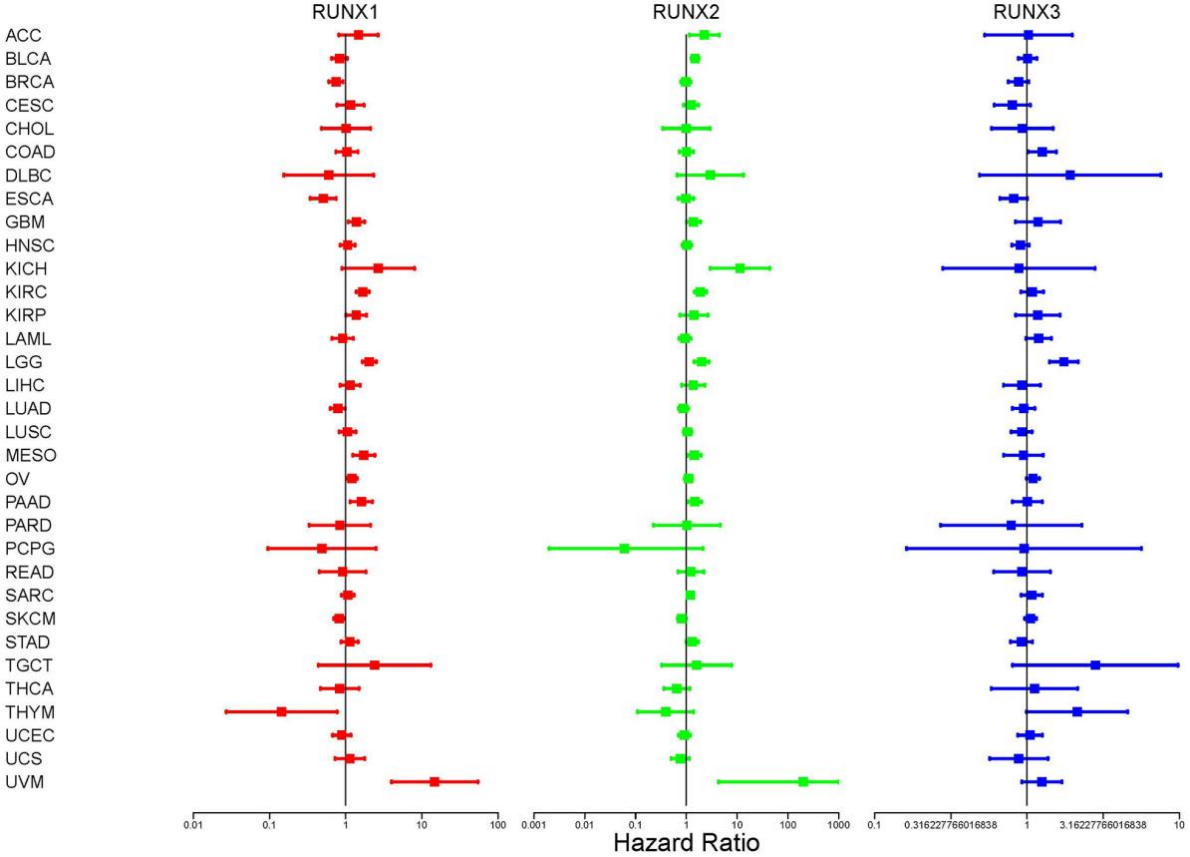
**Correlation analysis of *RUNX* gene family gene expression and patient survival by the Cox method in different cancer types.** Different colored lines represent the risk values of different genes in various cancers; HR<1 represents low risk and HR>1 represents high risk. Univariate Cox proportional hazard regression models were used for the association tests.

We used Cox analysis to look at the relationship between RUNX gene family expression and patient prognosis across different tumor types. According to our results, all *RUNX* gene family members showed different expressions associated with the prognosis of patients. Specifically, RUNX1 had a detrimental role in UVM, LGG, MESO, KIRC, PAAD, GBM, KIRP, and OV (HR>1, *p*<0.05); however, it had a protective role in SKCM, LUAD, BRCA, ESCA, and THYM (HR<1, *p*<0.05). RUNX2 was detrimental in UVM, KICH, ACC, LGG, KIRC, BLCA, PAAD, MESO, GBM, and SARC (HR>1, *p*<0.05) but protective in SKCM (HR<1, *p*<0.05). Furthermore, greater RUNX3 expression was linked to worse survival outcomes in LGG and COAD (HR>1, *p*<0.05, Figure 3). Detailed results of the Cox analysis of the *RUNX* gene family across the TCGA database are summarized in Supplementary Table 1.

**Figure 4:**
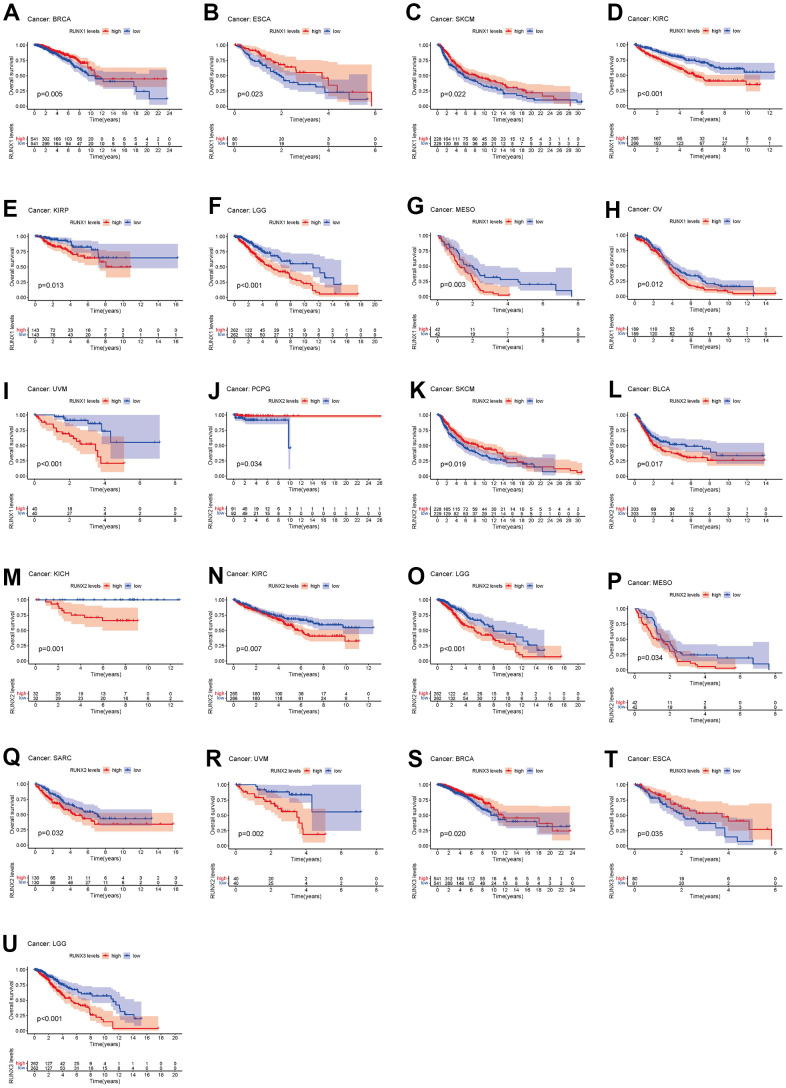
**Kaplan–Meier survival curves comparing pan-cancer high and low expression of *RUNX* gene family genes.** OS survival curves for RUNX1 in different cancers: (**A**) BRCA, (**B**) ESCA, (**C**) SKCM, (**D**) KIRC, (**E**) KIRP, (**F**) LGG, (**G**) MESO, (**H**) OV, (**I**) UVM. OS survival curves for RUNX2 in different cancers: (**J**) PCPG, (**K**) SKCM, (**L**) BLCA, (**M**) KICH, (**N**) KIRC, (**O**) LGG, (**P**) MESO, (**Q**) SARC. OS survival curves for RUNX3 in different cancers: (**R**) UVM, (**S**) BRCA, (**T**) ESCA, (**U**) LGG.

We utilized Kaplan–Meier survival curves to assess patient OS based on clinical data retrieved from TCGA in order to better understand the RUNX gene family’s predictive significance. Of the three family members, RUNX1 predicted the best prognosis in patients with BRCA, ESCA, and SKCM (all *p*<0.05, Figure 4A–4C). By contrast, in patients with KIRC, KIRP, LGG, MESO, OV, and UVM, RUNX1 indicated a poor prognosis (all *p*<0.05, Figure 4D–4I). In PCPG and SKCM, RUNX2 played a protective effect (all *p*<0.05, Figure 4J, 4K). On the other hand, RUNX2 was found to be harmful in seven cancer types: BLCA, KIRC, KICH, LGG, MESO, SARC, and UVM (all *p*<0.05, Figure 4L–4R). RUNX3 was shown to be beneficial in BRCA and ESCA (all *p*<0.05, Figure 4S, 4T) but a detrimental role in LGG (all *p*<0.05, Figure 4U).

**Figure 5:**
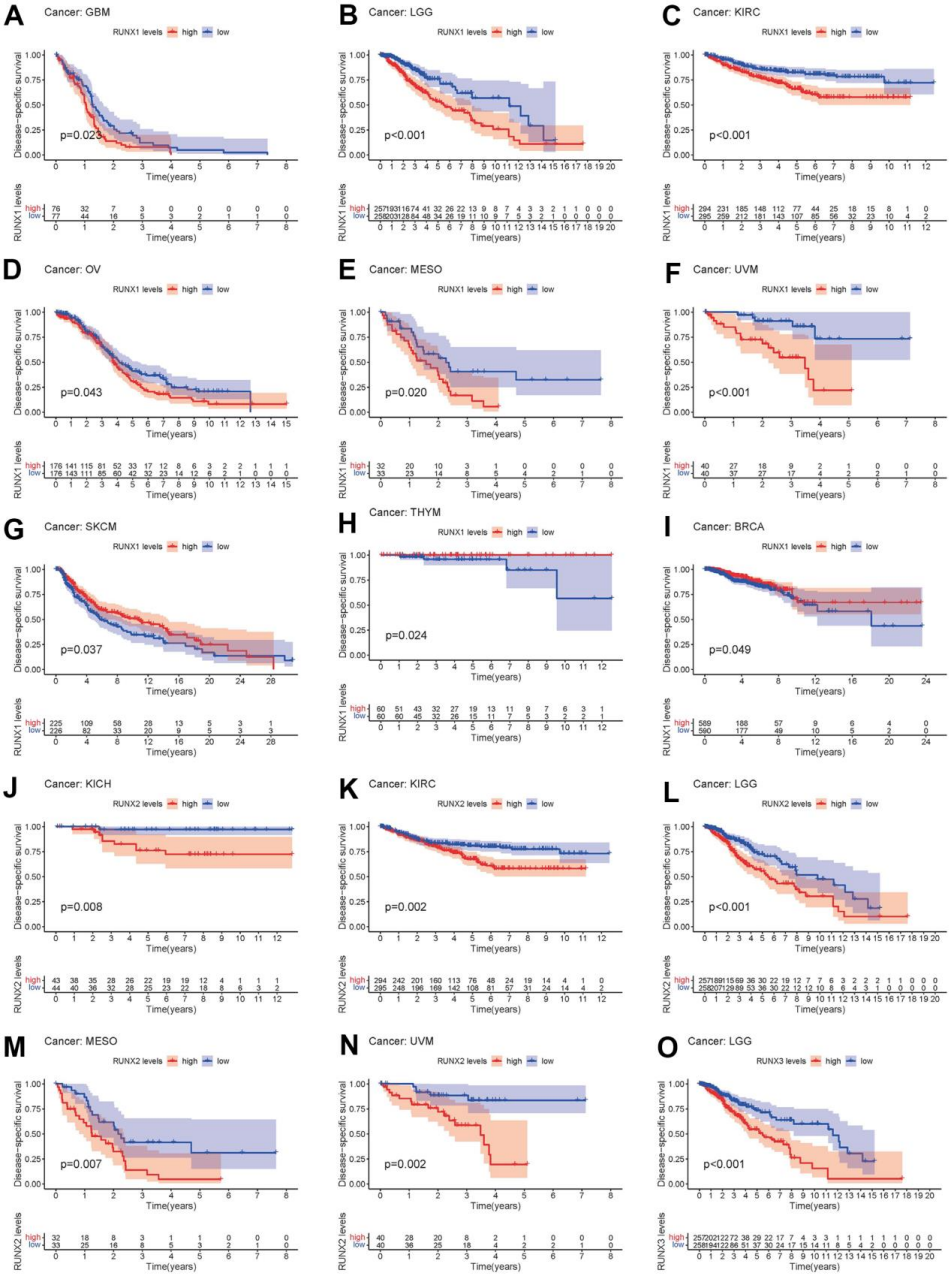
**Kaplan–Meier survival curves comparing pan-cancer high and low expression of *RUNX* gene family genes.** DSS survival curves for RUNX1 in different cancers: (**A**) GBM, (**B**) LGG, (**C**) KIRC, (**D**) OV, (**E**) MESO, (**F**) UVM, (**G**) SKCM, (**H**) THYM, (**I**) BRCA. DSS survival curves for RUNX2 in different cancers: (**J**) KICH, (**K**) KIRC, (**L**) LGG, (**M**) MESO, (**N**) UVM. DSS survival curves for RUNX3 in different cancers: (**O**) LGG.

We also examined the correlation between RUNX gene expression and DSS in pan-cancer. Higher RUNX1 expression was linked with poor DSS in GBM, LGG, KIRC, OV, MESO, and UVM, whereas increased RUNX1 expression predicted favorable DSS in SKCM, THYM, and BRCA (all *p*<0.05, Figure 5A–5I). RUNX2 played a negative influence in KICH, KIRC, LGG, MESO, and UVM (all *p*<0.05, Figure 5J–5N). RUNX3 was shown to be harmful in LGG (*p*<0.05, Figure 5O).

**Figure 6:**
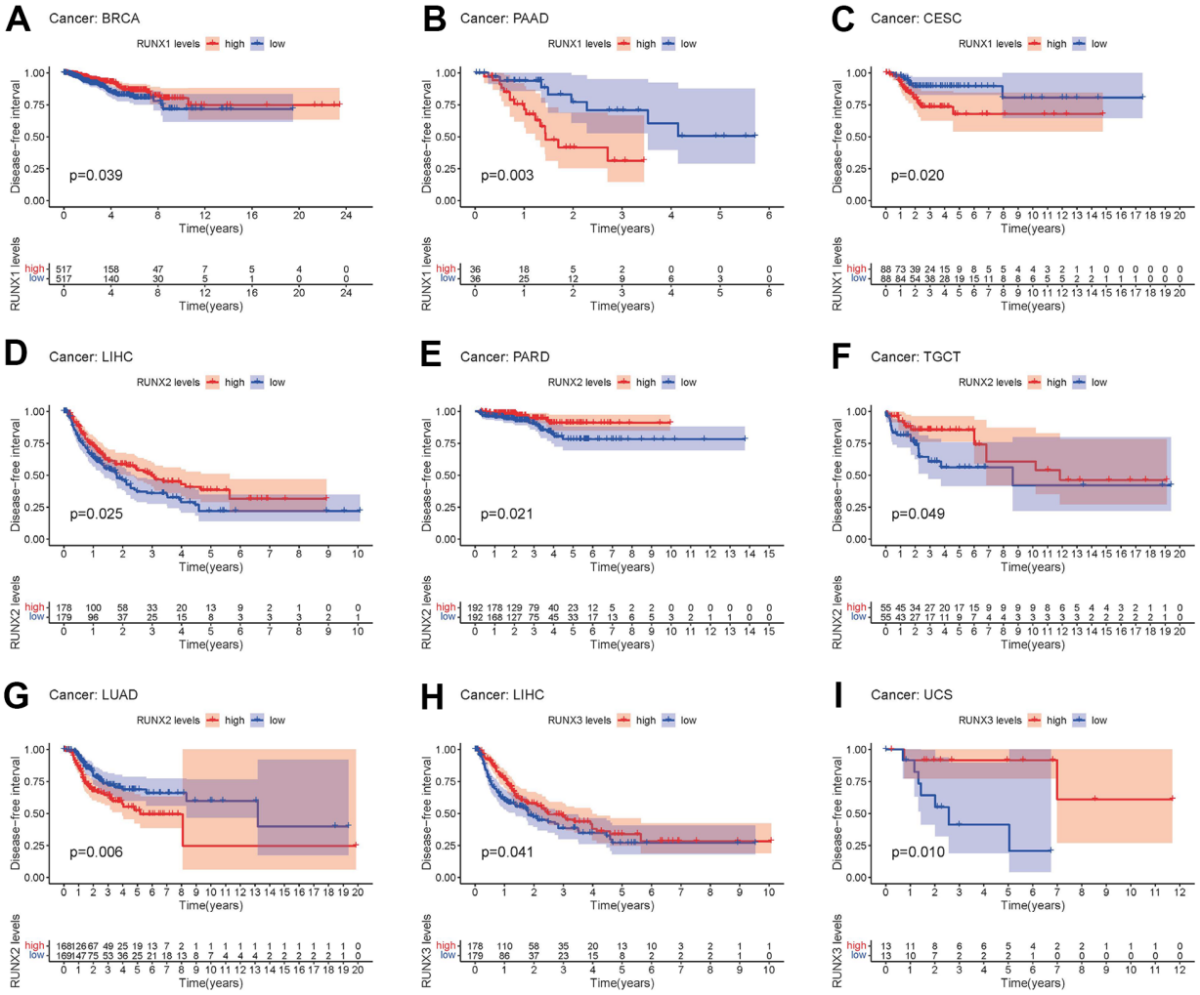
**Kaplan–Meier survival curves comparing pan-cancer high and low expression of *RUNX* gene family genes.** DFI survival curves for RUNX1 in different cancers: (**A**) BRCA, (**B**) PAAD, (**C**) CESC. DFI survival curves for RUNX2 in different cancers: (**D**) LIHC, (**E**) PARD, (**F**) TGCT, (**G**) LUAD. DFI survival curves for RUNX3 in different cancers: (**H**) LIHC, (**I**) UCS.

DFI in 33 TCGA tumors is analyzed using the same way. RUNX1 played a protective function in BRCA (*p*<0.05, Figure 6A). On the other hand, RUNX1 had a negative impact in PAAD and CESC (all *p*<0.05, Figure 6B, 6C). In LIHC, PARD, and TGCT (all p<0.05, Figure 6D–6F), high RUNX2 expression was a protective factor, while it was all a risk factor in the LUAD (all *p*<0.05, Figure 6G). In LIHC and UCS, RUNX3 played a protective function (*p*<0.05, Figure 6H, 6I).

**Figure 7:**
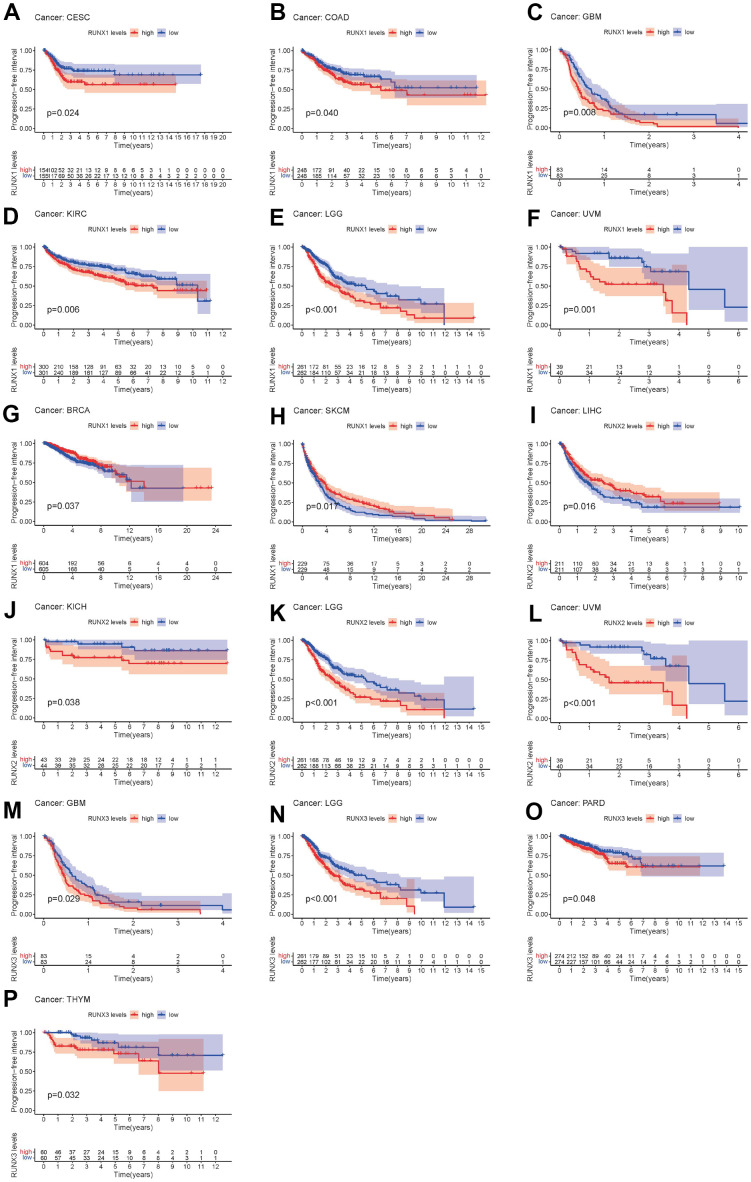
**Kaplan–Meier survival curves comparing pan-cancer high and low expression of *RUNX* gene family genes.** PFI survival curves for RUNX1 in different cancers: (**A**) CESC, (**B**) COAD, (**C**) GBM, (**D**) KIRC, (**E**) LGG, (**F**) UVM, (**G**) BRCA, (**H**) SKCM. PFI survival curves for RUNX2 in different cancers: (**I**) LIHC, (**J**) KICH, (**K**) LGG, (**L**) UVM. PFI survival curves for RUNX3 in different cancers: (**M**) GBM, (**N**) LGG, (**O**) PARD, (**P**) THYM.

Finally, the PFI was examined in 33 TCGA cancers. RUNX1 expression had a detrimental role in CESC, COAD, GBM, KIRC, LGG, and UVM (all *p*<0.05, Figure 7A–7F), but was all a protective role in the BRCA and SKCM (all *p*<0.05, Figure 7G, 7H). The high expression of RUNX2 was a protective factor in LIHC (all *p*<0.05, Figure 7I), but was all a risk factor in the KICH, LGG, and UVM (all *p*<0.05, Figure 7J–7L). RUNX3 had a detrimental role in GBM, LGG, PARD, and THYM (*p*<0.05, Figure 7M–7P).

We also examined the correlation between the expression of the *RUNX* gene family and the clinicopathologic stage. The expression levels of the *RUNX* gene family in multiple tumor tissues were considerably varied in different clinical phases, according to our findings. RUNX1 expression rose in the UVM as tumor grade increased, while RUNX2 expression increased in the STAD, ESCA, and KICH. In contrast, when tumor grade grew, RUNX1 expression in BLCA and BRCA declined, and RUNX3 expression in TGCT decreased (Supplementary Figure 1A–1G).

### Correlation of *RUNX* gene family expression with tumor microenvironment and tumor stemness

**Figure 8:**
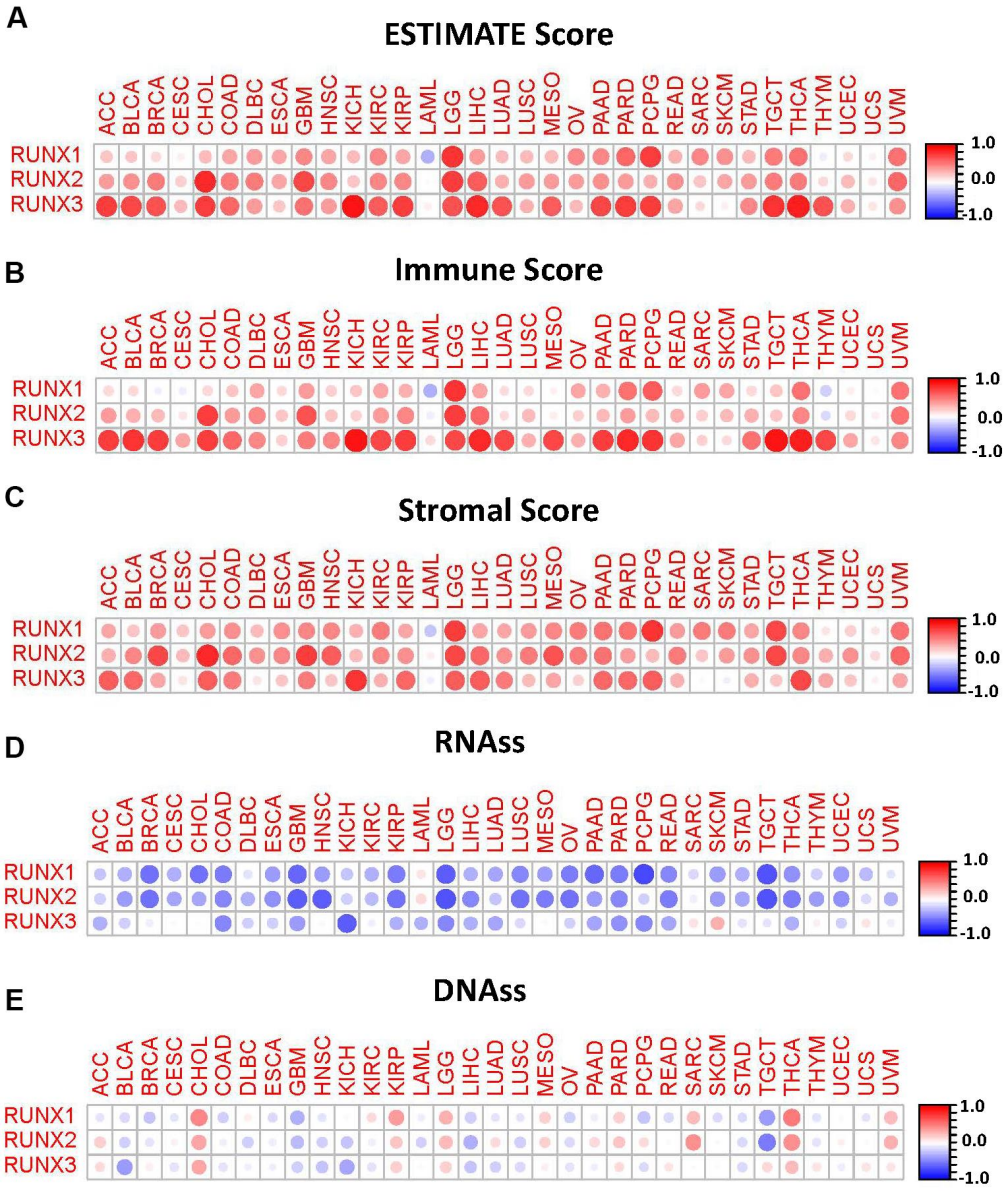
**Correlation matrix plots of *RUNX* gene family gene expression with tumor microenvironment and stemness scores in 33 different cancer types.** (**A**–**C**) RUNX gene family gene expression was associated with ESTIMATE, immune, and stromal scores in different cancers. (**D**, **E**) *RUNX* gene family gene expression was associated with RNAss and DNAss in different cancers. Spearman’s correlation was used for testing. Red dots indicate a positive correlation between gene expression and immune/stromal score, and blue dots indicate a negative correlation. The size of each dot represents the absolute value of the correlation coefficient.

The tumor microenvironment plays a crucial part in the initiation, progression metastasis, and drug resistance of cancer [17, 18]. We used the ESTIMATE method to generate immune and stromal scores, as well as tumor purity, in a variety of cancer types from the TCGA database to investigate the relationship between the *RUNX* gene family and the tumor microenvironment. We discovered a favorable pan-cancer correlation between the *RUNX* gene family and ESTIMATE scores after a series of investigations (Figure 8A–8C). This shows that the *RUNX* gene family’s three members have comparable roles in the tumor microenvironment.

In addition, we assessed the pan-cancer correlations between the *RUNX* gene family and tumor stemness. Using the TCGA tumor stemness database, RNAss and DNAss were analyzed. Our results showed that the *RUNX* gene family had a negative correlation with RNAss (Figure 8D). By contrast, it had a positive correlation with DNAss in CHOL, KIRP, LGG, MESO, THCA, and UVM (Figure 8E).

We also analyzed the relationship between TMB or MSI and the RUNX gene family expression in various malignancies. RUNX1 expression was strongly related with TMB in MESO, LUSC, LIHC, LGG, LAML, KIRP, HNSC, ESCA, CESC, BRCA, and BLCA, among the three family members (Supplementary Figure 2A). In LUSC, LUAD, KIRC, HNSC, DLBC, and CHOL, we discovered that gene expression was substantially linked with MSI (Supplementary Figure 2B). RUNX2 expression was shown to be connected with TMB in many malignancies, including LIHC, KIRP, KICH, HNSC, ESCA, CHOL, and BRCA (Supplementary Figure 2C), whereas RUNX2 expression was found to be correlated with MSI in LIHC, HNSC, DLBC, and COAD (Supplementary Figure 2D) (Supplementary Figure 2D). In PAAD, LUAD, LIHC, LGG, KIRP, ESCA, and BRCA, RUNX3 expression was shown to be substantially linked with TMB (Supplementary Figure 2E). Gene expression was also shown to be strongly linked with MSI in OV, LGG, KIRP, ESCA, DLBC, COAD, and BRCA in OV, LGG, KIRP, ESCA, DLBC, COAD, and BRCA (Supplementary Figure 2F).

### Correlation of RUNX gene family expression with tumor immune subtype and immune checkpoints

**Figure 9:**
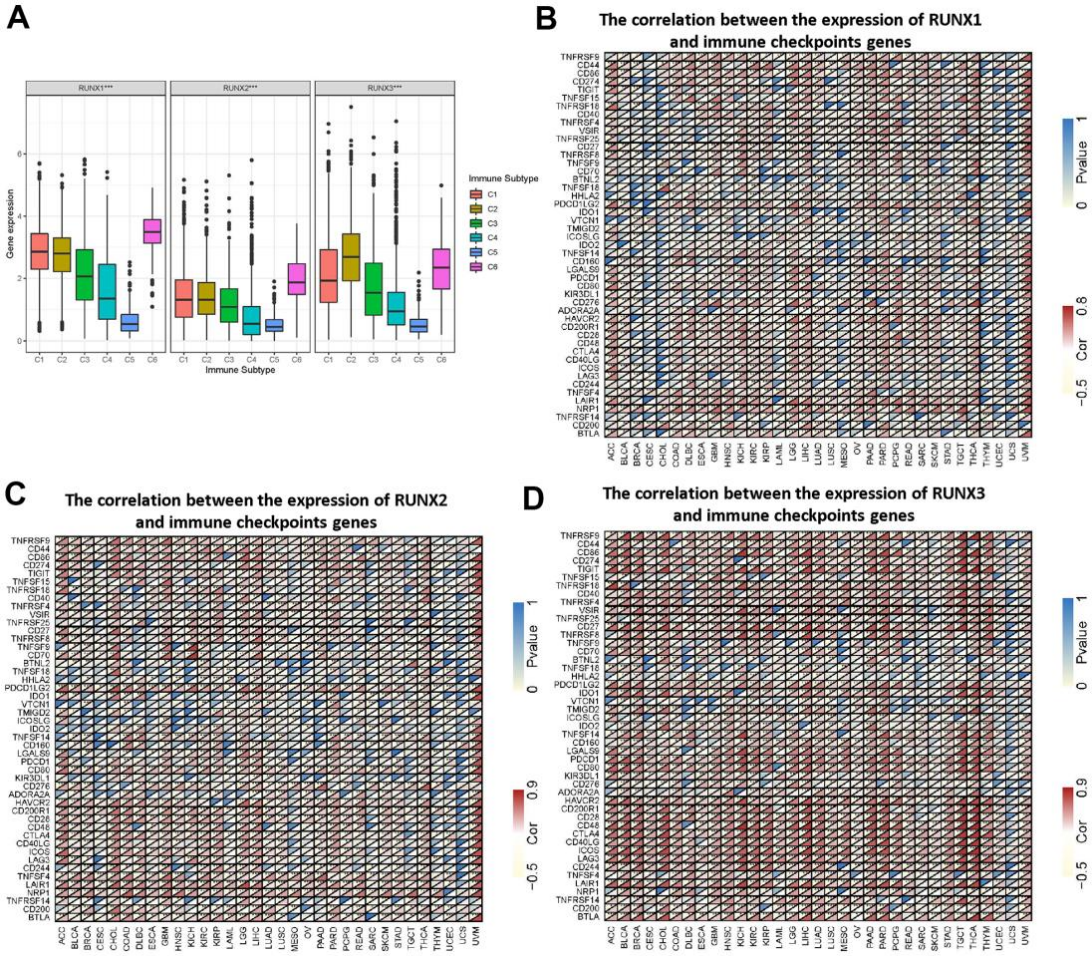
**Association of *RUNX* gene family gene expression with immune infiltrate subtypes and immune checkpoints.** (**A**) Association of *RUNX* gene family gene expression with immune infiltrate subtypes across all types of cancer (*p*<0.001) tested with ANOVA. X-axis represents immune subtype; Y-axis represents gene expression. (**B**–**D**) The correlations between RUNX1 (**B**), RUNX2 (**C**), and RUNX3 (**D**) and confirmed immune checkpoints in multiple cancers (*p < 0.05, **p < 0.01, ***p < 0.001).

Tumor-infiltrating immune cells are critical elements of the tumor microenvironment and assist malignancy elude the immune system. We investigated the possible association between the expression of the *RUNX* gene family and tumor immune subtypes to better understand the molecular signature of the *RUNX* gene family in tumor-infiltrating immune cells. On a pan-cancer basis, members of the *RUNX* gene family were all linked to immunological subtypes. RUNX1 expression was greater in C1, C2, C3, C4, and C6. RUNX2 levels were found to be higher in C1, C2, and C6. RUNX3 was found to be more expressed in C1, C2, C3, and C6 cells (Figure 9A). We also looked at the relationships between the *RUNX* gene family and 47 frequent immune checkpoint genes in the TCGA database across all cancer types. RUNX1 expression was substantially linked with immunoinhibitory genes in most malignancies, except CESC, CHOL, MESO, LAML, and UCS, as shown in Figure 9B. RUNX2 expression was also highly linked with immunostimulatory genes, with the exception of CESC, MESO, and UCS (Figure 9C). Except for UCS, RUNX3 expression was substantially linked with immunostimulatory genes (Figure 9D). This showed a possible *RUNX* gene family synergy with known immunological checkpoints.

### Genetic alteration analysis of *RUNX* gene family

**Figure 10:**
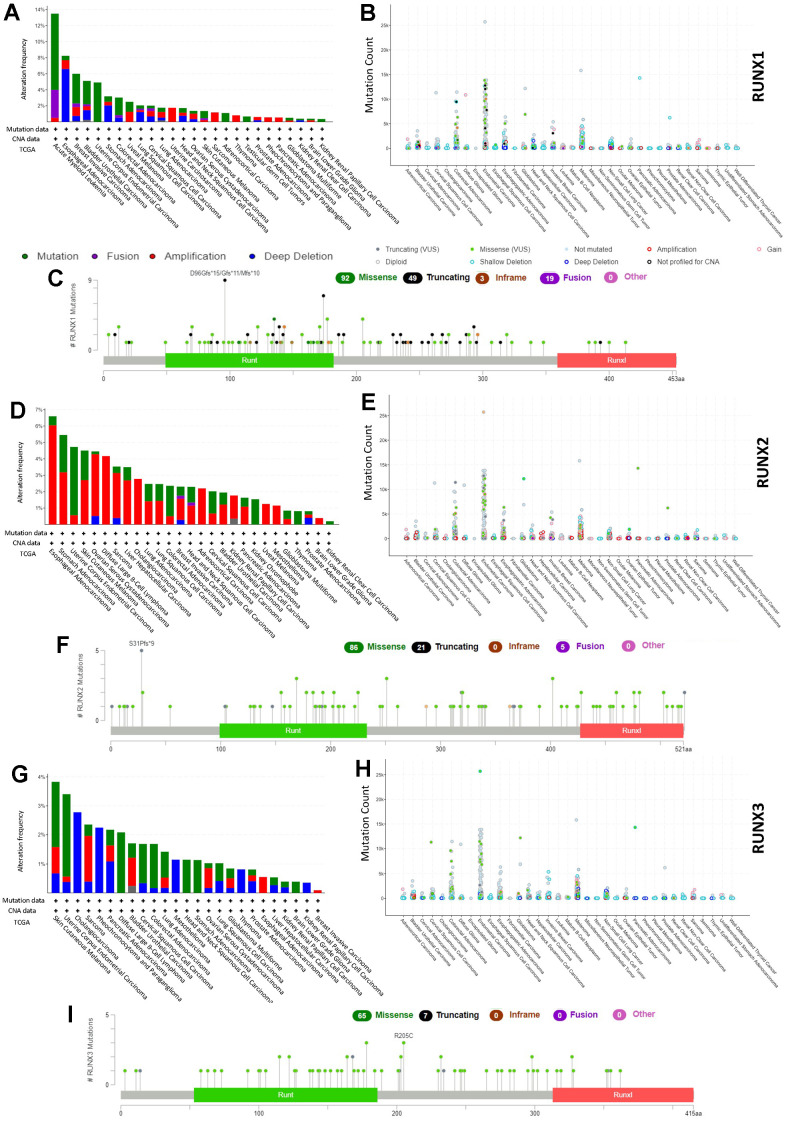
***RUNX* gene family gene mutation landscape.** The mutation frequency (**A**), general mutation count (**B**), and mutation diagram (**C**) of RUNX1 in multiple TCGA pan-cancer studies according to the cBioPortal database. The mutation frequency (**D**), general mutation count (**E**), and mutation diagram (**F**) of RUNX2 in multiple TCGA pan-cancer studies according to the cBioPortal database. The mutation frequency (**G**), general mutation count (**H**), and mutation diagram (**I**) of RUNX3 in multiple TCGA pan-cancer studies according to the cBioPortal database.

Using the cBioPortal database (10967 samples from 32 studies), we analyzed the genetic alterations of the *RUNX* gene family in different cancer types. Our results show that LAML had a relatively high mutation level, with RUNX1 alterations exceeding 13% with “mutation” as the primary type; the hot spot mutation of RUNX1 was D96Gfs*15/Gfs*11/Mfs*10 in the Runt domain, which occurred in nine cancers (LAML and BRCA) in nine patients and resulted in a truncated protein (Figure 10A–10C). The highest alteration frequency of RUNX2 (>6%) appeared for patients with esophageal adenocarcinoma with primary type “amplification” (Figure 10D, 10E). The RUNX2 hotspot mutation S31Pfs*9 resulted in a shortened protein and was found in five malignancies (STAD and COAD) in five individuals (Figure 10F). RUNX3 has a modest amount of genetic change when compared to RUNX1 and RUNX2. Patients with SKCM had the highest prevalence of RUNX3 mutations, with “mutation” being the most common form (Figure 10G–10I).

### Drug response analysis of *RUNX* gene family

**Figure 11:**
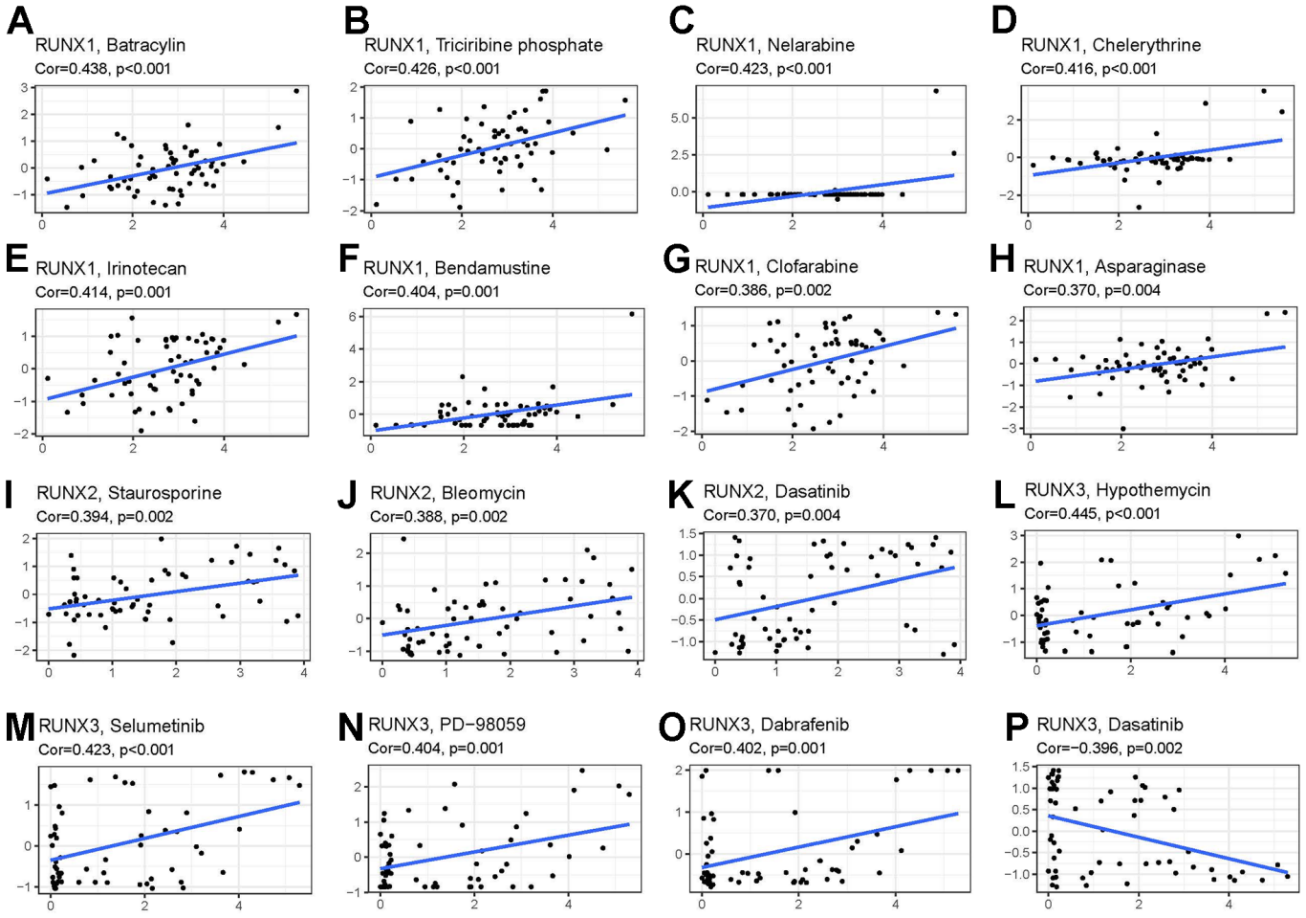
**Drug sensitivity analysis of *RUNX* gene family gene.** RUNX1 expression was positively associated with sensitivity to (**A**) batracylin, (**B**) triciribine phosphate, (**C**) nelarabine, (**D**) chelerythrine, (**E**) irinotecan, (**F**) bendamustine, (**G**) clofarabine, and (**H**) asparaginase; RUNX2 expression was positively related to sensitivity to (**I**) staurosporine, (**J**) bleomycin, and (**K**) dasatinib; and RUNX3 expression was positively associated with sensitivity to (**L**) hypothemycin, (**M**) selumetinib, (**N**) PD-98059, and (**O**) dabrafenib and negatively related to sensitivity to (**P**) dasatinib.

We used a correlation analysis to screen out the association between the *RUNX* gene family and drug response in various human cancer types from the CellMiner database to study the potential relationship between the *RUNX* gene family and drug response in various human cancer types. The *Z*-score was employed to assess medication response in this investigation. RUNX1 expression was found to be linked to a good response to batracylin, tricirbine phosphate, nelarabine, chelerythrine, irinotecan, bendamustine, clofarabine, and asparaginase (Figure 11A–11H). The expression of RUNX2 was related to a good response to staurosporine, bleomycin, and dasatinib (Figure 11I–11K). Hypothemycin, selumetinib, PD-98059, dabrafenib, and dasatinib were all linked to RUNX3 expression (Figure 11L–11P).

### Functional enrichment analysis of the *RUNX* gene family

**Figure 12:**
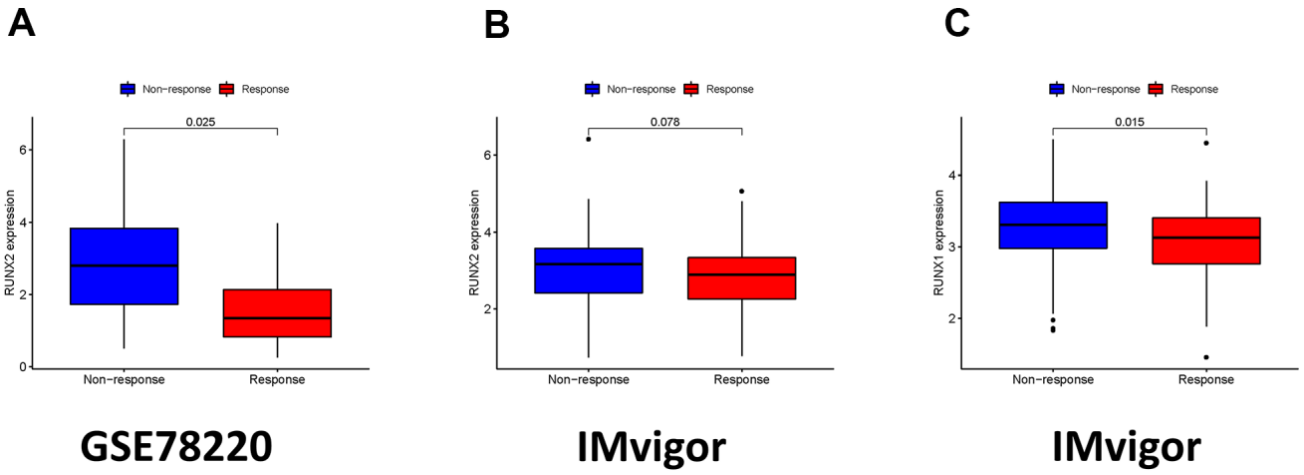
**The role of ICI scores in the prediction of immunotherapeutic benefits.** (**A**) RUNX2 expression in groups with a different anti-PD-1 clinical response status in the GSE78220. (**B**) RUNX2 expression in groups with a different anti-PD-L1 clinical response status in the IMvigor210 cohort. (**C**) RUNX1 expression in groups with a different anti-PD-L1 clinical response status in the IMvigor210 cohort.

We utilized GeneMANIA to screen out other genes or transcription factors linked with the *RUNX* gene family in order to do a full functional and pathway analysis of the *RUNX* gene family. Several transcription factors (Figure 12) were identified as possible targets of *RUNX* gene family members. RUNX members and related transcription factors were also found to be involved in the following pathways or functions: chromatin, regulatory region DNA binding, regulatory region nucleic acid binding, histone deacetylase binding, transcriptional repressor complex binding, and activating transcription factor binding, according to the study.

### *RUNX* gene family in immunotherapy

We also explored the connection between *RUNX* gene family expression and therapeutic benefit in patients who had anti-PD-1 and anti-PD-L1 immunotherapy. In the GSE78220 cohort, the low expression of RUNX2 group had a greater objective response rate than the high expression of RUNX2 group (*p* = 0.025; Figure 12A). The IMvigor cohort, which received anti-PD-L1 treatment, had a similar result (*p* = 0.075; Figure 12B). In the IMvigor210 cohort, we also discovered that decreased RUNX1 expression is associated to objective response to anti-PD-L1 treatment (*p* = 0.015; Figure 12C). Overall, these findings suggest that ICI scores may be linked to immunotherapy responsiveness.

## DISCUSSION

Several research published in the last several years have attributed the RUNX gene family to tumor development, invasion, metastasis, and treatment response [19]. However, no evidence of a link between RUNX gene family members and different cancers has been found in a pan-cancer study. For the first time, we used TCGA, Oncomine, and cBioPortal to analyze the RUNX gene family (RUNX1, RUNX2, and RUNX3) in 33 distinct human cancer types and matched normal tissues. In summary, as compared to comparable normal tissues, all three members of the RUNX gene family demonstrated considerable aberrant expression in diverse forms of cancer. RUNX1 was found to be overexpressed in 14 cancer types and low expressed in one. RUNX2 had higher expression in 12 types of cancer and low expression in one type. RUNX3 was overexpressed in nine types of cancer and showed low expression in five types. These findings imply that members of the RUNX gene family might be used as tumor biomarkers. Furthermore, we discovered considerable variation in RUNX gene expression across various tumor types in the TCGA. The functional complexity of the RUNX gene family, as well as the variety of tumors, might explain these contradicting pan-cancer gene expression levels. However, further research is needed to better understand the RUNX gene family’s possible function.

The current study looked at the link between the *RUNX* gene family and patient survival in 33 distinct cancer types. The predictive roles of members of the *RUNX* gene family differed among cancer types, according to our findings. In KIRC, LGG, MESO, and UVM, RUNX1 and RUNX2 were found to be strongly linked to poor prognosis. Furthermore, RUNX3 played a negative influence in LGG. RUNX1 and RUNX3 were, on the other hand, strongly linked to a better prognosis in BRCA and ESCA, while RUNX1 and RUNX2 were linked to enhanced survival in SKCM. However, increased RUNX3 expression has been linked to a poor prognosis in gastric cancer in multiple investigations [20, 21]. These contradictory results and inconsistent data for RUNX3 are due to the use of different data collection methods and the different roles of RUNX3 biological characteristics. More molecular experimental and clinical research is needed to investigate whether high expression of RUNX3 has an essential role in gastric cancer. We also found that the expression of the *RUNX* gene family was significantly correlated with the clinicopathologic stage in various tumors. We assumed that the discrepancy between our online database findings and the provided data was due to the varied methodologies used to assess RUNX expression. In summary, these results suggest that members of the *RUNX* gene family are potential prognostic markers for various cancers.

We also explored at the connections between the *RUNX* gene family and the tumor microenvironment as well as tumor stemness. The tumor microenvironment has been demonstrated in several studies to enhance tumor spread and tumorigenesis [22–24]. The *RUNX* gene family was linked to several tumor-stromal and immunological scores, as well as tumor purity, using the ESTIMATE methodology. Previous investigations have partially confirmed these conclusions. RUNX1 has been linked to the proliferation and differentiation of mesenchymal stem cells into myofibroblasts [25]. RUNX3/TGF-β1 were also related to the cancer-associated fibroblasts in the tumor microenvironment, which can increase promote tumor progression and lead to poor prognosis in colorectal cancer [26]. In prostate cancer, activation of RUNX2 promotes tumor microenvironment modification, which promotes carcinogenesis and disease progression [27]. These findings indicate that the *RUNX* gene family could have synergistic effects on immune functions in cancers. Mechanistic studies will be needed in the future to validate the relationship between *RUNX* gene family expression and the tumor microenvironment at the cellular and molecular levels. In addition, we analyzed the correlations between these genes and tumor stemness scores. Our results showed that the *RUNX* gene family has a clear relationship with RNAss and DNAss. Previous studies have indicated that higher RNAss and DNAss scores were related to tumor dedifferentiation and tumor metastasis, indicating potential drug targets for tumor chemotherapy [28, 29]. The findings suggest that RUNX expression may impact cancer patients’ responses to immune checkpoint therapy, which will help researchers better understand how immunotherapy works in cancer treatment.

Members of the RUNX gene family have been found to have critical roles in immune response and immune cell infiltration in previous investigations. In the LUAD immunological microenvironment, RUNX3 has been found to increase CD8+ T cell recruitment [30]. We looked at the links between the RUNX gene family and immunological subtypes to better understand the link between the RUNX gene family and immune cell infiltration. Patients with the C4 and C6 immunological subtypes had a dismal prognosis, according to previous research [31]. All members of the RUNX gene family, including C1, C2, and C6, were linked to more aggressive immunological subtypes, according to our findings. Members of the RUNX gene family were found to be more strongly expressed in C3, C4, and C5 in UVM. Previous research has found a link between C4 and macrophages [32]. Expression of RUNX3 can also promote monocyte-macrophage differentiation in metastatic melanoma [33]. These findings could help clinicians to identify patients who respond to such immunotherapies. However, the relationships between *RUNX* gene family members and immune infiltration need more extensive and in-depth research to fully explain them.

The possible association between the RUNX gene family and treatment response, according to the CellMiner database, was another noteworthy feature of this study. CellMiner is a database and query tool for cancer researchers that makes it easier to integrate and analyze genetic and pharmacological data for the NCI-60 cancer cell lines. Both p53-null and p53-mutated pancreatic cancer cells become more sensitive to gemcitabine when RUNX2 is depleted [34]. RUNX3 overexpression in gastric cancer cells can make them more sensitive to chemotherapeutic medicines, whilst its downregulation can make them more resistant to several treatments [35]. To create novel anticancer medication methods, substantial research is undoubtedly required. Overall, we discovered that the RUNX gene family’s gene expression levels were associated to treatment responsiveness, indicating that members of this family may be involved in drug resistance.

There were several limitations to this investigation. It started with a bioinformatic study to show RUNX gene family profiles in human malignancies, including expression, prognostic value, and mutation status; there were no *in vivo* or *in vitro* investigations to back up these findings. As a result, future research should concentrate on the mechanism of the RUNX gene family in various cancers.

In summary, we obtained a comprehensive and systematic pan-cancer description of the *RUNX* gene family. Our results indicate that the *RUNX* gene family is associated with levels of immune infiltration, poor prognosis, and drug response in various types of cancer. In addition, these findings highlight the potential use of members of the *RUNX* gene family as biomarkers for cancer progression and prognosis, and as potential treatment targets in various cancer types [36–39].

## Supplementary Material

Supplementary Figures

Supplementary Table 1
