# Supplementary material for: Prognostic value and immune characteristics of RUNX gene family in human cancers: a pan-cancer analysis
Source: Aging (Albany NY). 2022 May 6;14(9):4014–35. doi: 10.18632/aging.204065 (PMC9134966; doi:10.18632/aging.204065)
Supplement: Supplementary Figures [file aging-14-204065-s001.pdf]

SUPPLEMENTARY FIGURES

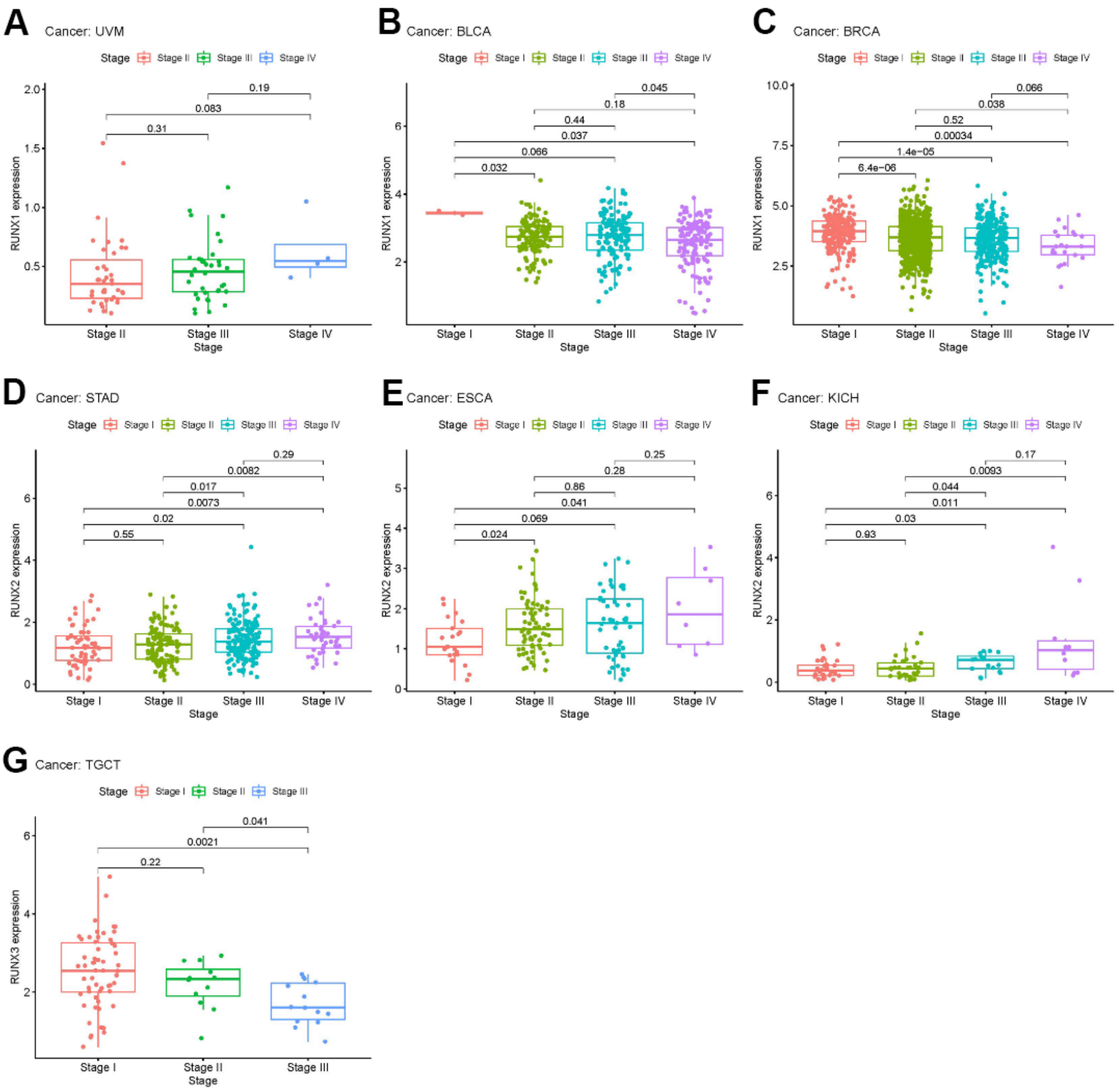

Supplementary Figure 1. The relationship between the *RUNX* gene family expression and clinicopathologic stage in seven tumors. (**A**) UVM; (**B**) BLCA; (**C**) BRCA; (**D**) STAD; (**E**) ESCA; (**F**) KICH; (**G**) TGCT.

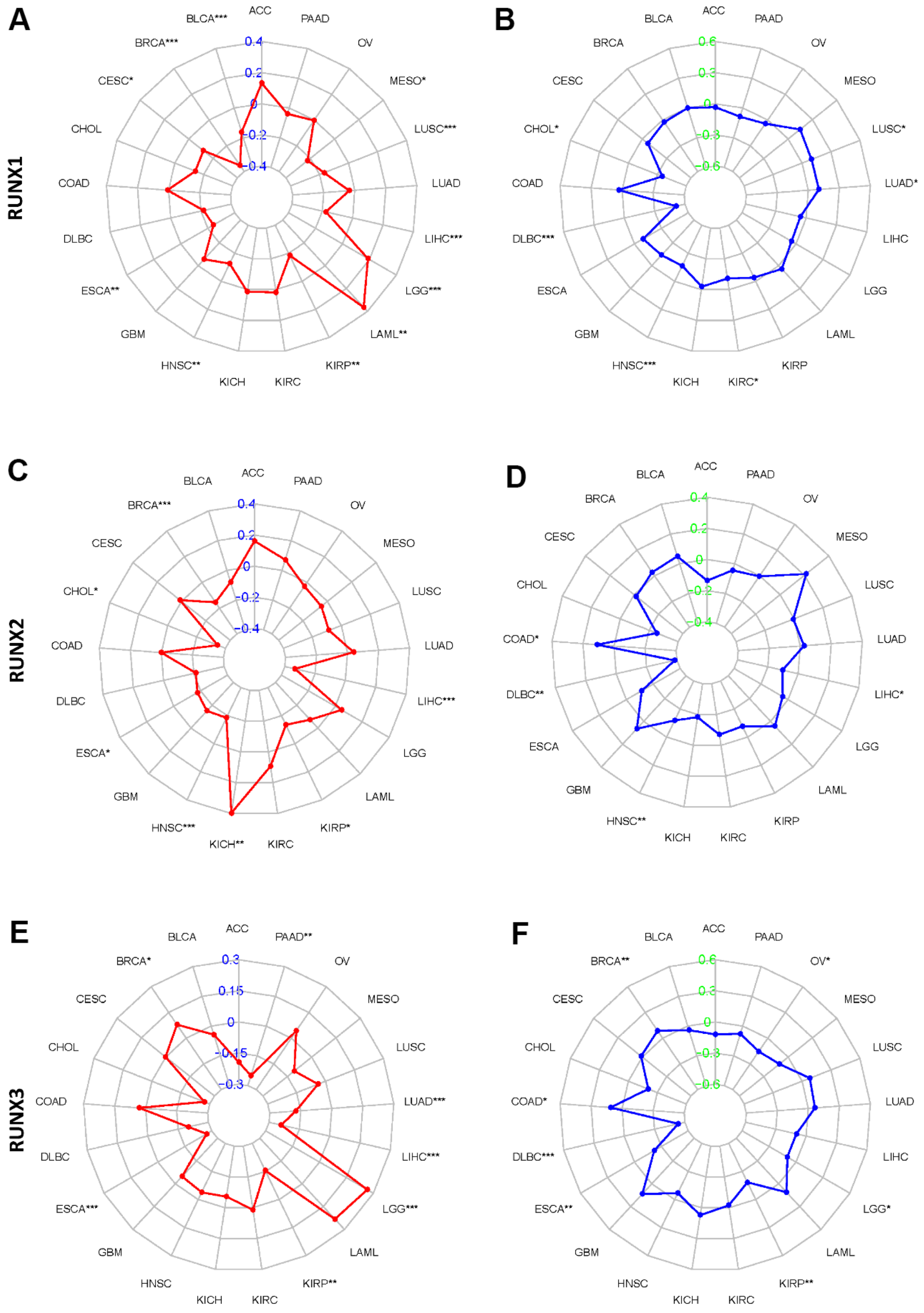

Supplementary Figure 2. The correlation of the *RUNX* gene family expression with tumor mutation burden (TMB) and microsatellite instability (MSI) in multiple cancer. (**A**) Correlation between TMB and RUNX1 expression. (**B**) Correlation between MSI and RUNX1 expression. (**C**) Correlation between TMB and RUNX2 expression. (**D**) Correlation between MSI and RUNX2 expression. (**E**) Correlation between TMB and RUNX3 expression. (**F**) Correlation between MSI and RUNX3 expression.
