# Supplementary material for: Prognostic value and immune characteristics of RUNX gene family in human cancers: a pan-cancer analysis
Source: Aging (Albany NY). 2022 May 6;14(9):4014–35. doi: 10.18632/aging.204065 (PMC9134966; doi:10.18632/aging.204065)
Supplement: Supplementary Table 1 [file aging-14-204065-s002.pdf]

SUPPLEMENTARY TABLE

Supplementary Table 1. The *RUNX* gene family was correlated with the prognosis outcomes of various types of cancer by COX analysis.

Gene	Cancer	HR	HR.95L	HR.95H	*p*-value
RUNX1	UVM	14.75821	4.000897	54.43899	5.30E-05
	LGG	2.04245	1.65557	2.519739	2.64E-11
	MESO	1.72881	1.239648	2.410995	0.001256
	KIRC	1.673365	1.377189	2.033236	2.22E-07
	PAAD	1.602605	1.144623	2.243831	0.006022
	GBM	1.385909	1.081422	1.776128	0.009926
	KIRP	1.376692	1.010592	1.875416	0.042687
	OV	1.207139	1.031108	1.413222	0.019238
	SKCM	0.82253	0.69727	0.970293	0.020464
	LUAD	0.786363	0.626963	0.986289	0.037579
	BRCA	0.743775	0.600874	0.920661	0.006541
	ESCA	0.505512	0.34117	0.749018	0.000673
	THYM	0.144182	0.026928	0.771997	0.023683
RUNX2	UVM	200.8339	4.311879	9354.218	0.006817
	KICH	11.43438	2.952063	44.28934	0.000421
	ACC	2.262608	1.152218	4.443079	0.017717
	LGG	1.98839	1.425085	2.774357	5.25E-05
	KIRC	1.896185	1.45008	2.47953	2.93E-06
	BLCA	1.484209	1.25217	1.759246	5.30E-06
	PAAD	1.464	1.082175	1.980544	0.01343
	MESO	1.444405	1.064438	1.960008	0.018229
	GBM	1.385374	1.009415	1.901359	0.043593
	SARC	1.188552	1.037704	1.361327	0.012616
	SKCM	0.813064	0.672936	0.98237	0.032012
RUNX3	LGG	1.745663	1.405269	2.168509	4.80E-07
	COAD	1.259565	1.017243	1.559611	0.034278
